# An Eco-friendly Iron Cathode Electro-Fenton System Coupled With a pH-Regulation Electrolysis Cell for p-nitrophenol Degradation

**DOI:** 10.3389/fchem.2021.837761

**Published:** 2022-01-28

**Authors:** Xiaohui Wang, Jingang Zhao, Chunyan Song, Xian Shi, Haipeng Du

**Affiliations:** ^1^ Technical Test Center of Sinopec Shengli OilField, Dongying, China; ^2^ Shengli Oilfield Testing and Evaluation Research Co., Ltd., SINOPEC, Dongying, China

**Keywords:** pH-regulation, electrolysis, iron cathode, electro-fenton, salt

## Abstract

The high consumption of salt reagents and strict pH control are still bottlenecks for the full-scale application of the Fenton reaction. In this work, a novel eco-friendly iron cathode electrochemical Fenton (ICEF) system coupled with a pH-regulation divided electrolysis cell was developed. In a pH-regulation divided electrolysis system, the desired pH for an effective Fenton reaction and for a neutral treated media could be obtained by H_2_O splitting into H^+^ and OH^−^ at the anode and cathode, respectively. In an ICEF system, an iron plate was used as the cathode to inhibit the release of iron ions and promote the reduction of Fe^3+^ to Fe^2+^. It was found that when a potential of 1.2 V/SCE was applied on the iron cathode, 98% of p-nitrophenol was removed in the combined system after 30 min with continuously adding 200 mg/L of H_2_O_2_. Meanwhile, a COD and TOC removal efficiency of 79 and 60% was obtained, respectively. In this case, the conductivity just slightly increased from 4.35 to 4.37 mS/cm, minimizing the increase of water salinity, as compared with the conventional Fenton process. Generally, this combined system was eco-friendly, energy-efficient, and has the potential of being a promising technology for the removal of bio-refractory organic pollutants from wastewaters.

## Introduction

Over the past few decades, advanced oxidation processes (AOPs) have attracted increasing interests for wastewater treatment since the highly oxidative hydroxyl radical (^•^OH, E^0^ = 2.80 V/SHE) was generated *in situ* and found to be capable of degrading any refractory organic molecules present in the aqueous solution until total mineralization at the kinetic constant values as high as 10^8^∼10^10^ M^−1^s^−1^ (Andreozzi et al., 1999; Oturan and Aaron, 2014; Gao et al., 2018). Among various AOPs, the conventional Fenton reaction process has been most widely applied for the treatment of wastewater streams because it exhibits the advantages of fast reaction rates, mild operating conditions, and simplicity to control (Bello et al., 2019). The Fenton reaction mainly proceeds via two steps (Neyens and Baeyens, 2003; Moreira et al., 2017; Gao et al., 2020). The first stage is characterized by the rapid formation of •OH from the homogeneous reaction between Fe^2+^ and H_2_O_2_ (Eq. 1), most of the pollutant degradation is achieved in this stage. The second stage is characterized by a slow reaction between Fe^3+^ and H_2_O_2_ for the regeneration of Fe^2+^ (Eq. 2), maintaining the continuous Fenton reaction (Babuponnusami and Muthukumar, 2014; Bello et al., 2019).

Fe2++ H2O2→ Fe3++OH-+O•H  k1= 63-76(L mol‐1s‐1) (1)

Fe3++H2O2→ Fe2++H•O2+ H+ k2= 0.001-0.01(L mol‐1s‐1) (2)

However, the Fenton process has some drawbacks, which greatly hamper its industrial application (Babuponnusami and Muthukumar, 2014; Gao et al., 2020). The addition of concentrated acid reagent is indispensable to adjust the solution pH to ∼3.0, that is the optimum condition for the Fenton reaction. However, working in such acidic pH requires the addition of a large amount of acid. In addition, massive alkaline reagents were also consumed for the subsequent neutralization of the treated solution. In addition, the employment of iron salt inevitably increases water salinity (Li et al., 2013). The increased salt content probably makes this wastewater unacceptable for natural environments or poses significant pressure on the subsequent reverse osmosis unit (Wang et al., 2014; Fang et al., 2018). Besides, most of the Fenton reagents are added at once while over-dosage of either H_2_O_2_ or iron ions would lead to side reactions (Eq. 3 and Eq. 4). As a result, Fe^3+^ is massively accumulated from the oxidation of Fe^2+^ in the Fenton reaction system since Fe^2+^ regeneration from Fe^3+^ is very slow (Qiang et al., 2003). Thus, the reduction of Fe^3+^ to Fe^2+^ greatly limits the treatment performance of the Fenton reaction for pollutants degradation.

Fe2++O•H→ Fe3++OH-   k3 = 3.2 ×108 (L mol‐1s‐1) (3)

O•H+H2O2→H•O2+ H2O   k4 = 3.3 × 107 (L mol‐1s‐1) (4)

Over the recent decades, there has been growing interest in the research community to address the above limitations of the conventional Fenton process. The development of a heterogeneous Fenton system has been demonstrated to be a feasible strategy to avoid salt reagents addition, where iron oxides and other metal oxides such as goethite (α-FeOOH), magnetite (Fe_3_O_4_), hematite (α-Fe_2_O_3_), and maghemite (γ-Fe_2_O_3_) have been commonly utilized as heterogeneous catalysts (Davarnejad and Azizi, 2016; He et al., 2016; Hassani et al., 2018; Görmez et al., 2019; Cai et al., 2021; Chen et al., 2021). Unlike the homogenous Fenton system, in a heterogeneous Fenton system, iron sources are immobilized within/on the catalyst structure, and the Fenton reaction occurs when the H_2_O_2_ molecule is in contact with the iron sites of the carrier, so that it is not necessary to continuously add the iron salts and pH limitation is reduced to some extent (Ganiyu et al., 2018; Cai et al., 2021; Hu et al., 2021). In spite of this, the most heterogeneous Fenton catalysts still operate optimally at pH 3–5 and its catalytic performance is reduced in near-neutral water bodies because the catalyst turnover frequency is reduced by up to 100-fold under neutral conditions (Cai et al., 2021; Chen et al., 2021).

Electro-Fenton is an emerging technology with well-known outstanding oxidation power, where the electron is utilized as the reagent to facilitate the regeneration of Fe^2+^ at the cathode (Eq. 5). Thus, this process exhibits higher performance in comparison with the conventional Fenton system because of the high utilization efficiency of Fe^2+^ (Oturan et al., 2000; Yuan et al., 2006; Brillas et al., 2009). Consequently, much more •OH can be produced at a much smaller amount of iron salt addition. In fact, the addition of iron salts can be completely avoided with using a metal iron plate as the sacrificial anode material, where the electrochemically released Fe^2+^ can serve as the Fenton catalyst. In spite of this, the produced ferric sludge amount cannot be controlled effectively. Besides, concentrated acid and basic reagents are still required to regulate the solution pH. In fact, in the typical electrolysis system, OH^−^ and H^+^ are produced at the cathode and anode, respectively, which can be separated well in the divided electrolysis cell. Inspired by this characteristic, the divided electrolysis cell can be used as the pH-regulation unit before and after the Fenton system to automatically acidify and neutralize the wastewaters without the requirement of chemicals. Besides, in the acid solution, the acidic dissolution of the metal iron plate can release the iron ions into the solution. Under the cathodic polarization of metal iron, the released iron ion amount can be well regulated. At the same time, Fe^3+^ can be also reduced to Fe^2+^ at the iron cathode. These collectively eliminate the limitations of the conventional Fenton process and promote its treatment efficiency for pollutants.

$${\text{Fe}}^{3+}{+\text{ e}}^{-}\to {\text{Fe}}^{2+}$$ (5)

In this study, a novel electro-Fenton process coupled with a pH-regulation divided electrolysis cell was developed for p-nitrophenol (PNP) degradation. In the pH-regulation divided electrolysis cell with a PTFE membrane as the separated material, the desired pH for an effective Fenton reaction and for a neutral treated media could be obtained by H_2_O splitting into H^+^ and OH^−^ at the anode and cathode, respectively. In the electro-Fenton process with external addition of H_2_O_2_, metal iron and a DSA electrode were applied as the cathode and anode, respectively, where the cathodic polarization of the metal iron electrode could effectively reduce the release of iron ions with diminishing ferric sludge generation.

## Experimental Section

### Materials

An iron plate (Fe, >99%) of 50 × 50 mm was purchased from Tengfeng Metallic Material Co. Ltd. (Hebei, China). p-nitrophenol (PNP, C_6_H_5_NO_3_, 99%) was supplied from Shanghai Yien Chemical Technology Co. Ltd. 1,10-phenanthroline (C_12_H_8_N_2_·H_2_O, 99%), glacial acetic acid (C_2_H_4_O_2_, 99%), sodium acetate (CH_3_COONa, 99%), ferrous sulfate (FeSO_4_.7H_2_O, 99%), hydroxylamine hydrochloride (HONH_3_Cl, 98.5%), sodium sulfate (Na_2_SO_4_, 99%), hydrogen peroxide (H_2_O_2_, 30%), titanium oxalate, potassium (K_2_TiO(C_2_O_4_)_2_, 98%), ferrous ammonium sulfate hexahydrate (Fe(NH_4_)_2_·(SO_4_)_2_·6H_2_O, 99%), potassium permanganate (KMnO_4_, 99%), sulfuric acid (H_2_SO_4_, 98%), and sodium hydroxide (NaOH, 98%) were provided by Sinopharm Chemical Reagent Co. Ltd., China. All chemicals were analytical grade and were used without further purification. Ultrapure water (18.2 MΩ cm) was used to prepare reaction solutions for all the experiments.

### Experimental Procedures

**FIGURE 1:**
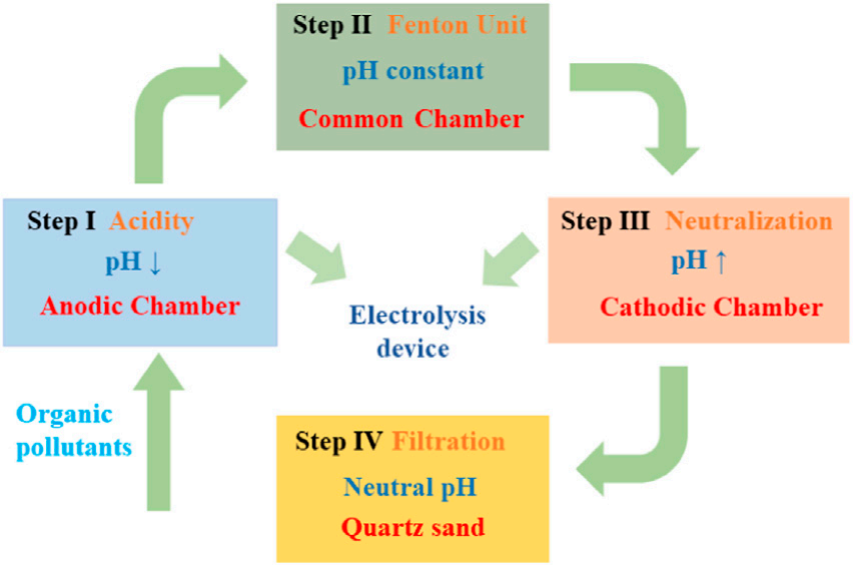
Scheme of the iron cathode electrochemical Fenton (ICEF) system (steps 2 and 4) coupled with the pH-regulation divided electrolysis system (steps 1 and 3).

As shown in Figure 1, the combined electrochemical treatment process mainly consisted of four steps. Prior to step 1, a 100 mg/L PNP solution with pH 7.0 was performed in the beaker, 3 g/L of Na_2_SO_4_ was used as the electrolyte solution. In step 1, pH adjustment was carried out in the divided electrolysis cell, which used a plexiglass rectangular tank with the cathode and anode chambers isolated by a PTFE microfiltration membrane. The anode consisted of a 50 × 80 mm boron-doped diamond (BDD) electrode while the cathode consisted of titanium mesh of the same dimensions with the distance of 25 mm; the PTFE membrane was between the anode and cathode. The galvanostatic electrolysis reactions were performed by a DC power supply (voltage 0–30.0 V, electric current 0–5.0 A). In step 1, the PNP solution concurrently entered into the cathode and anode chambers, pH was reduced to near 3.0 in the anodic chamber within 1 min. In step 2, the solution with the reduced pH was added into the iron cathode electrochemical Fenton (ICEF) system for PNP degradation with a chamber volume of 500 ml using a three-electrode potentiostat (Model CHI1130C, Chenhua instrument Co. Ltd. Shanghai, China) coupled with a saturated calomel reference electrode (SCE), where the cathodic potential was precisely controlled by the potentiostat and SCE. The working electrode was a 50 × 50 mm iron plate, and the counter electrode was a 50 × 50 mm DSA (Ti-RuO_2_-IrO_2_) mesh; 200 mg/L of H_2_O_2_ was continuously added by a pump within 15 min. In step 3, after the treatment of PNP solution in the ICEF system, the solution was transferred into the cathode chamber of the divided electrolysis cell to increase the pH. In addition, the pH of the cathode effluent was further regulated to neutral (∼9.0) by NaOH solution. In step 4, the neutral solution was passed through a filter device to remove iron ions, which was carried out in a container filled with quartz sand (80–120 mm). The removal ratio of p-nitrophenol, COD, or TOC ( η,% ) was calculated via Eq. 8.

$$\text{η}=\;\frac{{\text{C}}_{0}-{\text{C}}_{\text{t}}}{{\text{C}}_{0}}\;\times 100\text{\%}$$ (6)

### Analytical Determinations

The concentrations of PNP were determined by high performance liquid chromatography (HPLC-1100, Agilent) with an Eclipse XDB-C18 column (4.6 mm × 150 mm, 5 µm), (Nakatsuji et al., 2015). The mobile phase was methanol/water (50/50), the flow rate was 1.0 ml/min, and the UV detector was set at 314 nm. The chemical oxygen demand (COD) was detected using a COD analyzer (ASH 6B-80, China) [37]. A total organic carbon analyzer (multi N/C® 2,100, Analytik Jena AG) was applied to monitor total organic carbon (TOC). (Kavitha and Palanivelu, 2004). The concentration of H_2_O_2_ was measured by the potassium titanium (IV) oxalate method at 400 nm with a UV-Vis spectrophotometer (UV 6000 PC Yuanxi instrument Co. Ltd. Shanghai, China) (Wang and Chu, 2011). Fe^2+^ and Fe (tot) were determined at 510 nm using a modified phenanthroline method with an Fe^2+^ detection limit of 0.5 μM, and Fe^3+^ concentration was estimated as the difference between Fe (tot) and Fe^2+^ (Xin et al., 2018). Solution pH and conductivity were measured by a water quality analyzer. The reaction was quenched in the collected samples by immediately adding 1.0 mol/L of NaOH since Fenton oxidation cannot occur at pH > 10.0. For COD measurements, the samples were pretreated with NaOH to remove any residual H_2_O_2_. All experiments were performed twice at least, with relative errors less than 3%.

## Results and Discussion

### pH-Regulation in a Divided Electrolysis Cell

**FIGURE 2:**
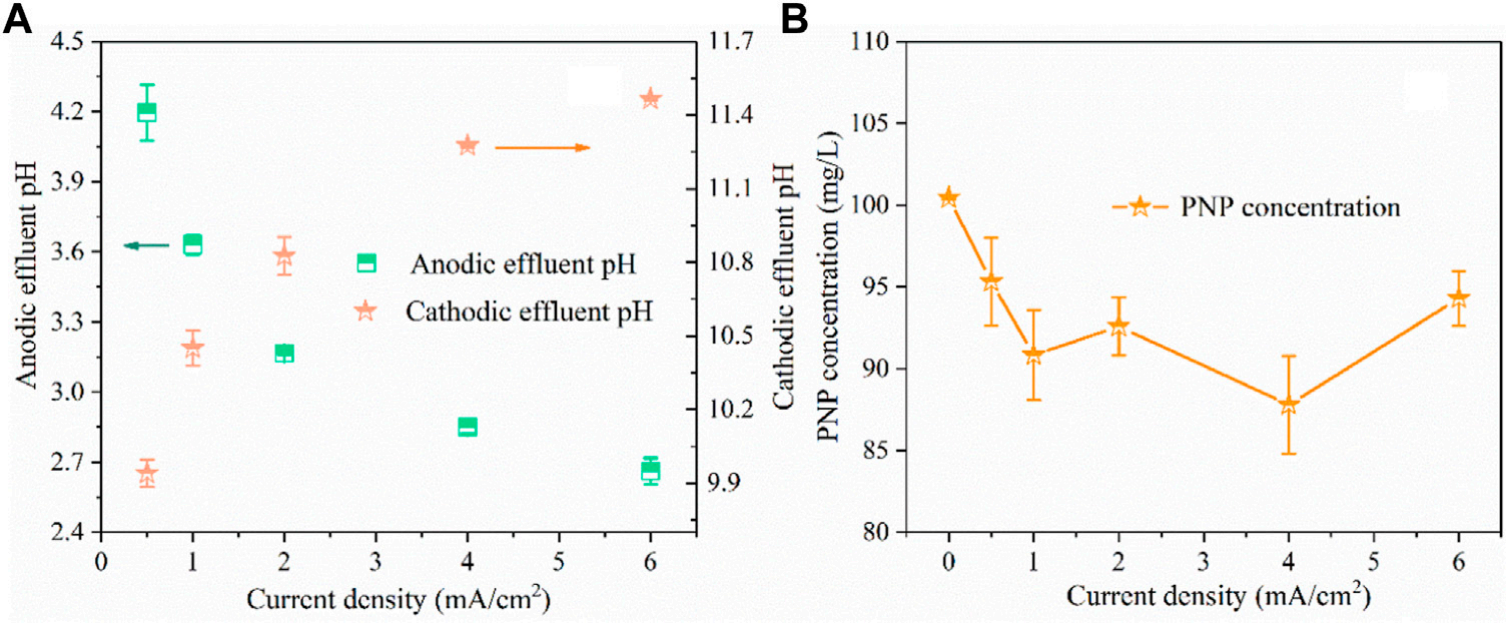
The influence of current density on effluent pH **(A)** and PNP concentration **(B)**. (PNP concentration = 100 mg/L, Na_2_SO_4_ = 3 g/L, pH_0_ = 7.0, anode compartment flow velocity = 20 ml/min, cathode compartment flow velocity = 20 ml/min).

For the Fenton reaction, it is recommended to acidify the solution pH to ∼3.0 for •OH production and then neutralize the solution for the precipitation of Fe^3+^ (Chen et al., 2021). In this study, we developed a divided electrolysis cell using a hydrophilic PTFE microfilter membrane as the separated material. In this system, H_2_O splitting reactions at the anode and cathode could produce H^+^ and OH^−^ (Eq. 7 and Eq. 8), respectively, which could be used to regulate the solution pH required for the Fenton reaction. Thus, the substrate solution pH could reduce to nearly 3.0 when it flowed through the anode chamber in step 1 (Figure 1). After the Fenton reaction, the solution pH was raised to promote the formation of Fe(OH)_3_ in step 3. The above pH adjustment objective could be achieved by regulating the applied current density and flow velocity. Here, a neutral solution containing 100 mg/L of PNP and 3 g/L of Na_2_SO_4_ electrolyte was pumped into the electrolysis device at a flow rate of 20 ml/min by a peristaltic pump, where the residence time was 1 min. The current density applied is the key parameter for the electrochemical pH regulation because it fundamentally affects the yield of H^+^ in the anode compartment and OH^−^ in the cathode compartment, respectively. As shown in Figure 2A, the pH of the anode effluent continuously decreased with the increase of current density; as the current density increased from 0.5 to 6 mA/cm^2^, the pH of anodic effluent decreased from 4.2 to 2.7, while the cathodic effluent pH increased from 9.9 to 11.5. This was consistent with what we expected, because as the current increased, effective water-splitting resulted in more H^+^ produced at the anode and more OH^−^ produced at the cathode. In particular, when the applied current density was 2 mA/cm^2^, an acidic effluent with pH of 3–3.2 was automatically attained at the steady state (Neyens and Baeyens, 2003). It has been demonstrated that the Fenton process is inhibited at extremely acidic environments due to the formation of (Fe(H_2_O))^2+^ and Fe(III)-hydroxyl complexes, Fe(OH)^2+^. Thus, to achieve the most favorable Fenton reaction condition, an applied current density of 2 mA/cm^2^ was adopted in this study.

$${2\text{H}}_{2}{\text{O }+\;2\text{e}}^{-}\to {2\text{OH}}^{-}{+\text{H}}_{2}↑$$ (7)

$${2\text{H}}_{2}{\text{O }-\;4\text{e}}^{-}\to {4\text{H}}^{+}{+\text{ O}}_{2}↑$$ (8)

The results in Figure 2B show that the PNP degradation efficiency increased as the current density increased initially from 0 to 1 mA/cm^2^, but it became nearly constant when the current density was elevated above 1 mA/cm^2^. Specifically, as the current density increased from 0.5 to 1 mA/cm^2^, the PNP removal efficiency increased from 5 to 10%. Nevertheless, further increasing the current density to 6 mA/cm^2^ did not increase the further removal of PNP. In this case, the BDD anode had little effect on the degradation of pollutants in this system because of a too short residence time.

### Iron Plate Immersed in Water Without Electricity

**FIGURE 3:**
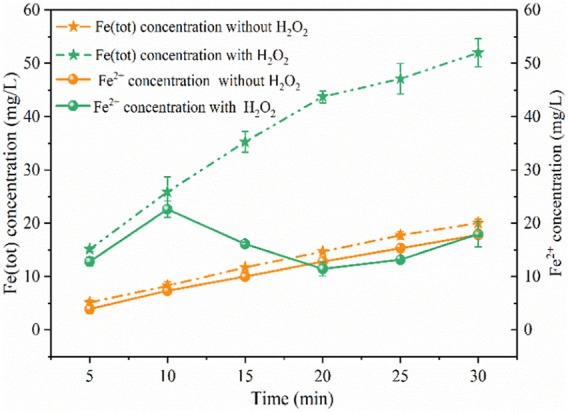
Concentration variations of iron ions under different conditions. (PNP = 100 mg/L, Na_2_SO_4_ = 3 g/L, pH_0_ = 3.0).

As shown in Figure 3, when the solution pH reduced to nearly 3.0, the iron plate immersed in water without power could produce 20 mg/L of Fe (tot) within 30 min, of which 89% was Fe(II). In this process, the iron plate would be dissolved by H^+^ to generate Fe^2+^ under acidic conditions through Eq. 9, and the oxidation of Fe^2+^ to Fe^3+^ by O_2_ was slow and the solution pH increased slightly from 3.0 to 3.2. The average production rate of Fe (tot) (approximately 0.33 mg/min) and Fe^2+^ (approximately 0.3 mg/min) was stable and closed within 30 min, indicating the stable release of Fe^2+^ by the iron plate under the acid solution. Under the same conditions, Fe (tot) significantly increased to 52 mg/L after 200 mg/L of H_2_O_2_ was continuously added into the solution within 15 min. The average production rate of Fe (tot) was stable within 20 min (approximately 1.1 mg/min), which was three times higher than that without the addition of H_2_O_2_. But these two reactions exhibited a similar average production rate of Fe (tot), approximately 0.41 mg/min, within the reaction period of 20–30 min, which may be ascribed to the fact that most of the H_2_O_2_ was consumed within 20 min. As for Fe^2+^ concentration, it continuously increased within 10 min with the production rate of approximately 1.13 mg/min and decreased from 23 to 11 mg/L between 10 and 20 min, which was explained by the consumption of Fe^2+^ during the Fenton reaction process (Eq. 1). And after 20 min, the concentration continuously increased, finally reaching 18 mg/L at 30 min, accounting for 35% of the Fe (tot). Within 20–30 min, the Fe (tot) concentration increased by 8 mg/L, close to the value of Fe^2+^ increase (7 mg/L), this can also be explained by the fact that little H_2_O_2_ remained after 20 min and the increase of Fe (tot) concentration was mostly due to the Fe^2+^ released (Eq. 9). The above results revealed that the presence of H_2_O_2_ could facilitate the release of iron ions from Fe^0^ in the acid solution. In this case, a large amount of iron sludge was produced and side reactions (Eq. 3) might consume produced •OH, leading to inferior degradation of pollutants.

$${\text{Fe}+2\text{H}}^{+}\to {\text{Fe}}^{2+}{+\text{H}}_{2}$$ (9)

### Effect of Different Potential Applied to Iron Plate Cathode in ICEF System

**FIGURE 4:**
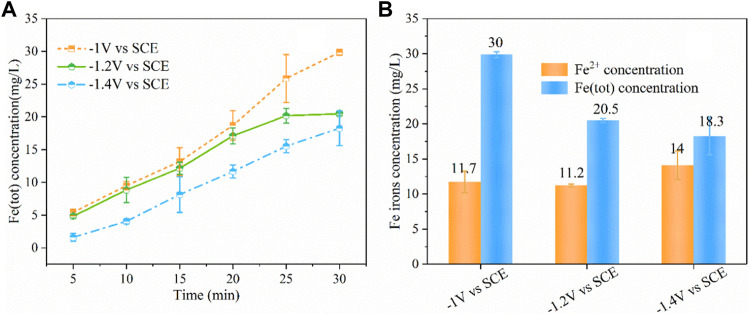
Concentration variations of Fe (tot) under different cathodic potentials **(A)**, concentration of Fe irons at 30 min under different cathodic potentials **(B)**. (PNP = 100 mg/L, Na_2_SO_4_ = 3 g/L, pH_0_ = 3.0).

To further reduce the release of iron ions from the iron cathode and improve the regeneration of Fe^2+^ from Fe^3+^, we developed a novel iron cathode electro-Fenton system, applying a potential on the iron cathode. Figure 4A shows that concentration variations of Fe (tot) under different cathodic potentials, it was found that as the applied cathodic potential increased, the produced Fe (tot) decreased continuously. At the cathodic potential of −1.0 and −1.2 V/SCE, the produced Fe (tot) concentration was 30 and 21 mg/L within 30 min, which reduced by 42 and 61%, respectively, compared with no electricity input. Further increasing the cathodic potential from −1.2 to −1.4 V/SCE resulted in the slight decrease of Fe (tot) concentration to 18 mg/L. The production rate of Fe (tot) (0.43 mg/min) at the cathodic potential of −1.2 V/SCE was significantly higher than −1.4 V/SCE (0.29 mg/min) within the initial 20 min, while the value was apparently decreased to 0.17 mg/min within 20–30 min, and the rate slightly increased to 0.33 mg/min during the same time at −1.4V/SCE. Figure 4B shows the Fe^2+^ and Fe (tot) concentrations produced at 30 min under different cathodic potentials. It was found that the ratio of Fe^2+^ to Fe (tot) concentration enhanced significantly from 0.39 to 0.77 with the decrease of cathodic potential from −1.0 to −1.4V/SCE. This can be explained by the fact that the regeneration from Fe^3+^ to Fe^2+^ increased with the decrease of cathodic potential.

**FIGURE 5:**
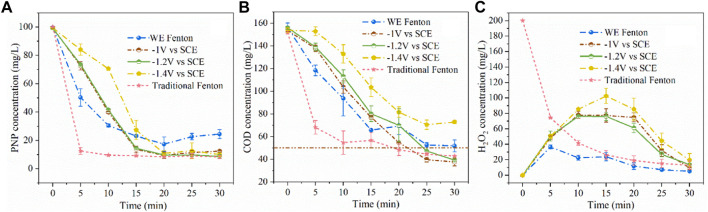
Concentration variations of PNP **(A)**, COD **(B)**, and H_2_O_2_ **(C)** during the degradation under different conditions (PNP = 100 mg/L, Na_2_SO_4_ = 3 g/L, pH_0_ = 3.0).

The results of PNP concentration versus time are shown in Figure 5A. It was found that only 75% of PNP was degraded within 30 min under the conditions of the iron plate immersed in water with 200 mg/L of H_2_O_2_ without electricity (WE Fenton process). And PNP removal efficiency was enhanced to 87, 91, and 90% at cathodic potentials of −1.0, −1.2, and −1.4 V/SCE, respectively (Kavitha and Palanivelu, 2004). However, it is noted that the removal efficiency was inferior initially then reached similar levels as the others after 20 min at −1.4 V/SCE. In addition, COD removal efficiency was basically the same in the case of −1 and −1.2 V/SCE, whereas the treatment at −1.4V/SCE exhibited an inferior degradation rate. As shown in Figure 5B, the COD removal efficiency was 67% without electricity, and increased to 76 and 75% at the cathodic potentials of −1.0 and −1.2 V/SCE, respectively, with residual COD concentration below 50 mg/L. However, when the cathodic potential was decreased to −1.4 V/SCE, the COD concentration reduced from 154 to 73 mg/L within 30 min, which can be ascribed to the inferior production of Fe (tot) for the Fenton reaction. According to the above results, −1.2 V/SCE was chosen as the optimal cathodic potential, and the released Fe (tot) concentration decreased by approximately 61% with the COD concentration further decreasing from 52 to 40 mg/L as compared with the WE Fenton process. The degradation of the 100 mg/L PNP solution was carried out in the traditional Fenton system with initially adding 20 mg/L of Fe^2+^ and 200 mg/L of H_2_O_2_ at pH_0_ 3.0. It was found that PNP was destructed quickly within 5 min and exhibited faster decay of PNP and COD than the present combined process within 20 min. However, its final COD removal efficiency was outperformed by the present combined process at 30 min and was slightly surpassed by the latter (Babuponnusami and Muthukumar, 2014).

**FIGURE 6:**
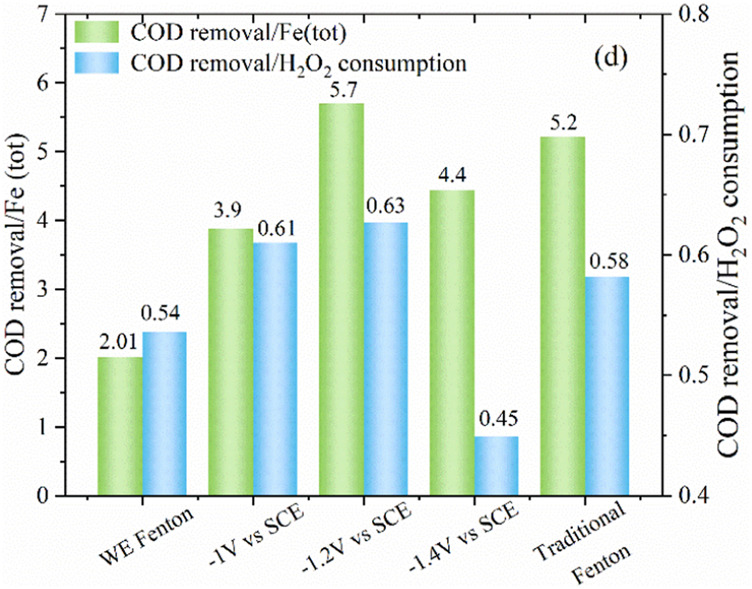
The ratio of COD removal to Fe (tot) concentration and H_2_O_2_ consumption under different conditions (PNP = 100 mg/L, Na_2_SO_4_ = 3 g/L).

The variation of H_2_O_2_ concentration is shown in Figure 5C. It was observed that when applying potential at the iron cathode, the H_2_O_2_ concentration increased initially within 15 min and then decreased afterwards in the late reaction stage. As compared with the WE Fenton reaction system, the H_2_O_2_ concentration was higher, which was probably due to the smaller release of iron ions from the iron cathode. As the applied cathodic potential decreased from −1.0 to −1.4 V/SCE, the remaining concentration of H_2_O_2_ was slightly higher over the whole reaction period, which was also associated with the production of iron ions. In the traditional Fenton system, the concentration of H_2_O_2_ significantly decreased from 200 to 126 mg/L within 5 min, which corresponded to the quick reaction between Fe^2+^ and H_2_O_2_, and the remaining H_2_O_2_ concentration at 30 min was 13 mg/L. From Figure 6, it could be clearly observed that as the cathodic potential decreased from −1.0 to −1.2 V/SCE, the ratio of COD removal to produced Fe (tot) and H_2_O_2_ consumption was increased from 3.9 to 5.7 and 0.61 to 0.63, while the value significantly decreased to 4.4 and 0.45 at a higher cathode potential of 1.4 V/SCE. These results demonstrated that -1.2 V/SCE was the optimal cathodic potential with suitable Fe (tot) production for the electro-Fenton reaction, which exhibited slightly higher COD removal/Fe (tot) and COD removal/H_2_O_2_ consumption for the traditional Fenton reaction system. In the WE Fenton process, the much lower value, i.e., 2 for COD removal/Fe (tot) and 0.54 for COD removal/H_2_O_2_ consumption, was obtained, respectively.

### The Divided Electrolysis Cell and ICEF Combined System

**FIGURE 7:**
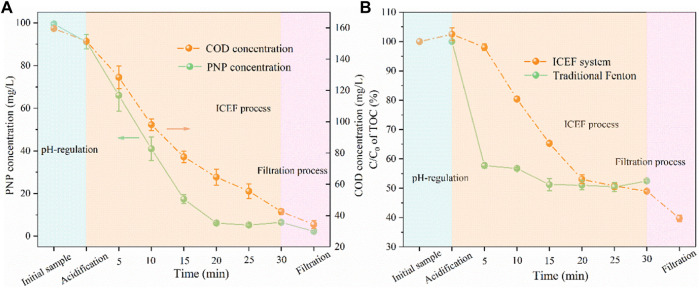
Concentration variations of PNP and COD **(A)** and TOC concentration reduction **(B)** during the EIC-EF system degradation (PNP = 100 mg/L, Na_2_SO_4_ = 3 g/L, pH_0_ = 7.0).

According to the aforementioned experimental results, the optimal current density for solution pH regulation and cathodic potential for the ICEF reaction have been obtained. The results of PNP and COD concentration time are shown in Figure 7A. It was found that the PNP concentration of the anodic effluent slightly decreased from 100 to 91 mg/L while the COD removal efficiency was only 5%, owing to the oxidation at the BDD anode. During the combined process, the removal efficiency of PNP and COD attained 93 and 72% within 30 min, and the PNP pollutant was further destructed after filtration because of the coagulation of iron ions with the concentration decreased from 6 to 2 mg/L. Figure 7B shows the TOC removal efficiency of traditional Fenton and ICFE systems, the removal efficiency was 42% within 5 min by traditional Fenton but maintained relatively stable in the remaining time, finally the value reached 48%. This can be explained by the rapid consumption rate of Fenton reagents. However, the TOC removal efficiency achieved 60% in the combined system, which was much higher than traditional Fenton (Kavitha and Palanivelu, 2004). The high removal efficiency of PNP (98%), COD (79%), and TOC (60%) revealed more efficient degradation of pollutants in the combined system than the traditional Fenton system.

**FIGURE 8:**
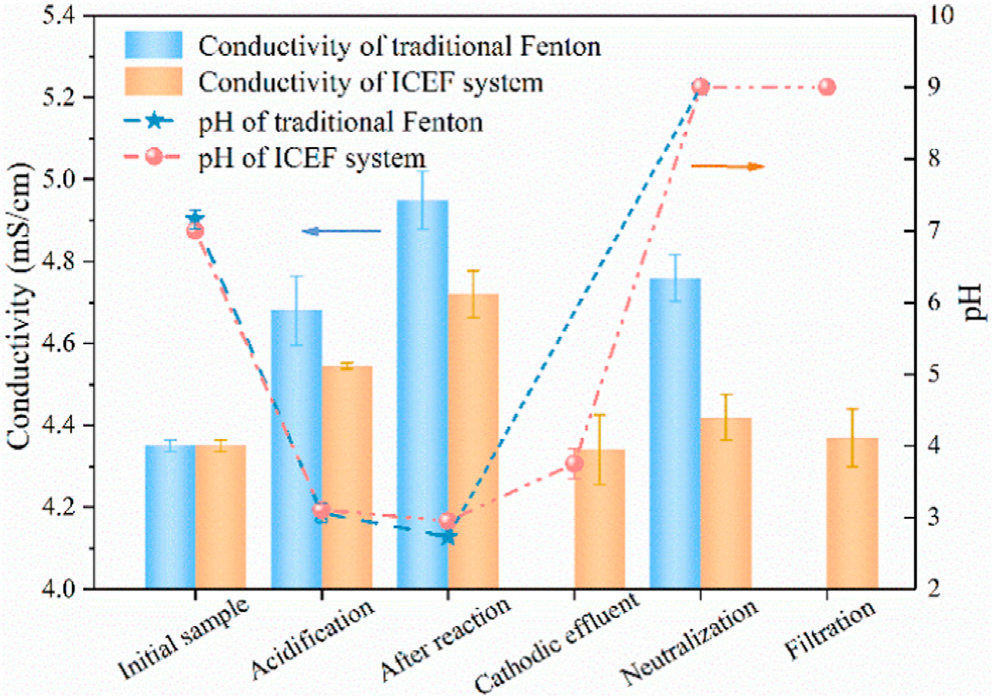
Concentration variations of conductivity and pH under different conditions. (PNP = 100 mg/L, Na_2_SO_4_ = 3 g/L).

For traditional Fenton, pH was adjusted to the desired values with H_2_SO_4_ and NaOH solution while ferrous sulfate was used as the iron ions source (Gao et al., 2020). As shown in Figure 8, the conductivity was increased from 4.35 to 4.68 mS/cm after acidification during traditional Fenton, and the value continuously enhanced to 4.95 after the Fenton reaction, while the pH slightly decreased from 3.07 to 2.73, which can be explained by the large quantity of organic acids production. The pH of the effluent was neutralized to approximately 9.0 with the conductivity reduced to 4.76 mS/cm, which might be caused by acid-base neutralization. As for the present combined process, the conductivity of the anodic effluent increased to 4.55 mS/cm with the pH decreasing from 7.0 to 3.1, the cathodic effluent pH only slightly increased from 2.95 to 3.75 at the same current density (2 mA/cm^2^), owing to the buffering effect of organic acids on pH. During the neutralization process, pH was further adjusted to neutral (∼9.0) by the NaOH solution (Chen et al., 2021), driving the complete precipitation of Fe(OH)_3_. In this case, the conductivity further decreased to 4.37 mS/cm. Generally, the salinity of traditional Fenton increased by 9% compared with the initial solution while the present combined process remained basically unchanged after the reaction, avoiding the obvious increase of water salinity caused by the addition of a large amount of chemical reagents in traditional Fenton.

### Mechanism Discussion

**FIGURE 9:**
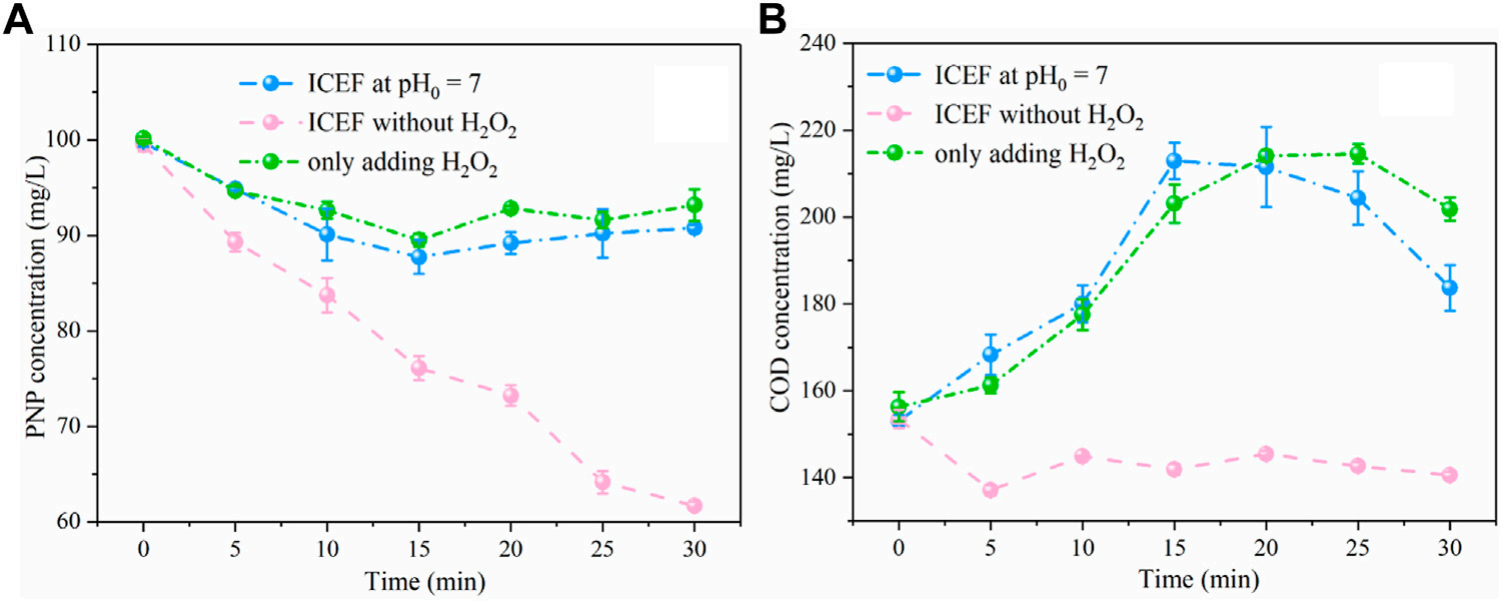
Concentration variations of PNP **(A)** and COD **(B)** under different conditions (PNP = 100 mg/L, Na_2_SO_4_ = 3 g/L).

A set of experiments was performed to verify the main mechanism of pollutant degradation. As shown in Figures 9A,B, the PNP removal efficiency was only 9% without front-end pH adjustment, owing to basically no Fe^2+^ production under the neutral solution, indicating that the pH-regulation process is indispensable for the ICEF system. In addition, only 7% of PNP was oxidized by H_2_O_2_ in solution. In the above two reaction system, COD concentration slightly increased after the reaction, which was possibly subject to formation of some intermediates which interfered with the COD concentration. The direct oxidation of PNP by the DSA anode was also examined without H_2_O_2_ at pH_0_ = 3.0, the removal efficiency of PNP and COD concentration were 38 and 13%, respectively. Thus, the direct PNP degradation at the anode was ruled out, indicating that PNP in solution was mainly oxidized by •OH produced by the Fenton reaction.

**FIGURE 10:**
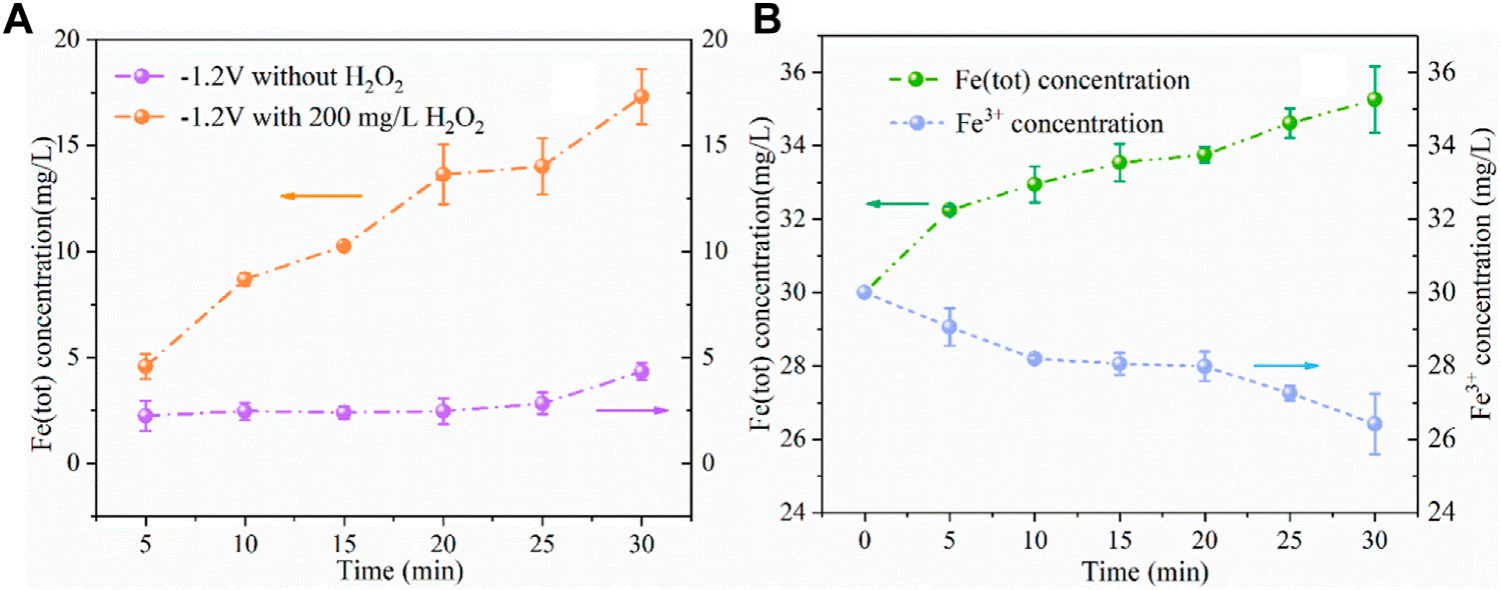
Concentration variations of Fe (tot) and Fe^2+^ **(A)** and Fe (tot) and Fe^3+^ **(B)** under different conditions (PNP = 100 mg/L, Na_2_SO_4_ = 3 g/L, pH_0_ = 3.0).

To further investigate the effect of cathode potential on iron ion concentration, we conducted the following experiments. As shown in Figure 10A, at the cathode potential of −1.2V/SCE without adding H_2_O_2_, the produced Fe (tot) concentration was 4 mg/L, which reduced by 80% compared with no electricity as shown in Figure 3 (20 mg/L). And in the case of adding 200 mg/L of H_2_O_2_ and applying the cathode potential of -1.2 V/SCE, the Fe (tot) concentration increased to 17 mg/L. There are two reasons for this phenomenon, one is that the iron plate would react with H_2_O_2_ in the presence of H^+^ through Eq. 10 (Pan et al., 2019); the other is that the presence of H_2_O_2_ promoted the conversion of Fe^2+^ to Fe^3+^ and the generated Fe^3+^ may further react with the iron plate to produce Fe^2+^ through Eq. 11. As shown in Figure 10B, 30 mg/L of Fe^3+^ was also added into the solution at the cathodic potential of -1.2 V/SCE without H_2_O_2_, the Fe (tot) concentration slightly increased to 35 mg/L within 30 min and Fe^3+^ concentration reduced to 26 mg/L. The results demonstrated that the reaction between Fe^3+^ and Fe^0^ contributed marginally to the release of iron ions during the ICEF process, while the direct oxidation of Fe^0^ by H_2_O_2_ probably dominated the release of iron ions from the iron plate. As a result, in the WE Fenton system, H_2_O_2_ was not only consumed for the Fenton reaction but also for the release of Fe^2+^ by the reaction with the iron plate. Thus, a part of H_2_O_2_ cannot be utilized for •OH production via the Fenton reaction, leading to the inferior degradation efficiency of PNP.

$${\text{Fe}+\text{H}}_{2}{\text{O}}_{2}{+\;2\text{H}}^{+}\to {\text{ Fe}}^{2+}{+2\text{H}}_{2}\text{O}$$ (10)

$${\text{Fe}}^{3+}+\text{ Fe}\to {\text{Fe}}^{2+}$$ (11)

### Environmental Implication

In the present combined system, the cost for electricity played an important role in the overall operating cost of the process. Apparently, the overall energy consumption is the sum of the pH adjustment unit and Fenton reaction process, the power consumption of per ton of wastewater was calculated based on Eq. 12 (Wang and Chu, 2011).

$$\text{E}=\;\frac{\text{UIT}}{\text{V}}\;\times 10^{-3}$$ (12)

where E is the energy consumption, U is the voltage measured during the reaction (V), I is the applied current (A), t is the electrolysis time (h), and V is the volume of reaction solution (m^3^).

**FIGURE 11:**
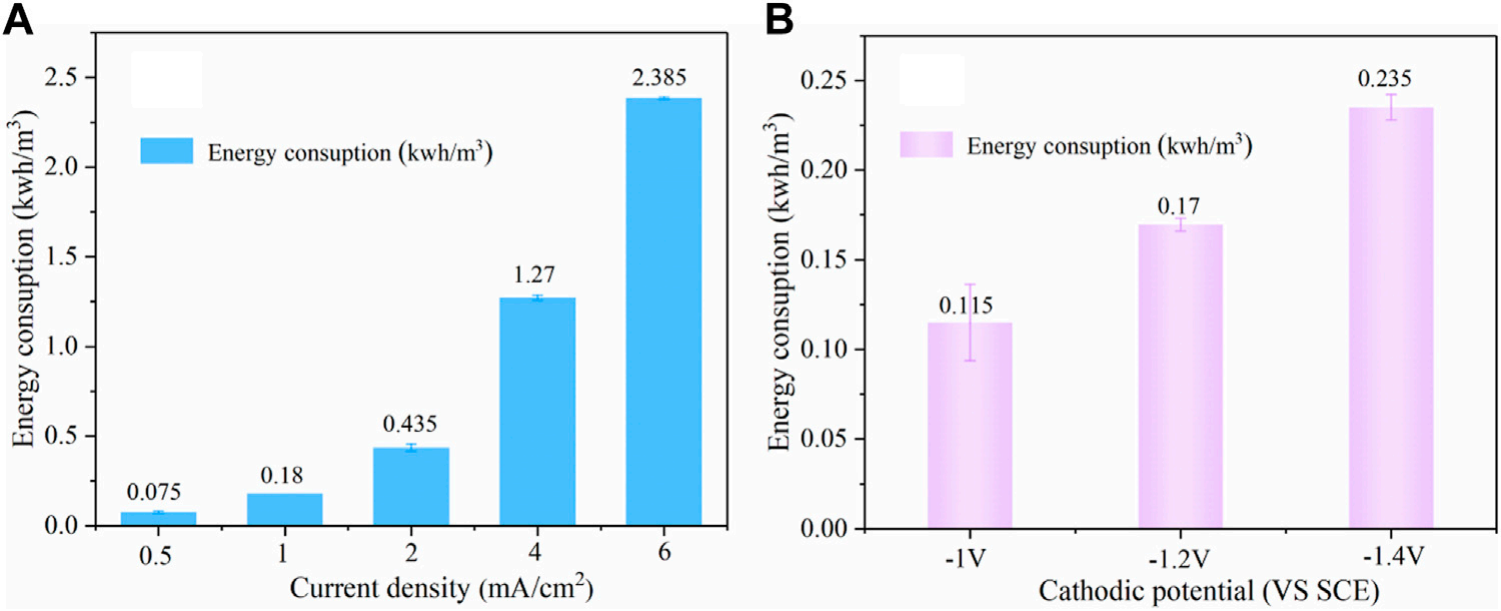
Effect of different current densities **(A)** and cathodic potential **(B)** on energy consumption (PNP = 100 mg/L, Na_2_SO_4_ = 3 g/L, pH_0_ = 7.0).

As shown in Figure 11A, during the pH-regulation process, current density had a significant impact on energy consumption, the value increased from 0.075 to 2.385 kWh/m^3^ as the current density increased from 0.5 to 6 mA/cm^2^. In the present combined system, a pH of 3.0–3.2 was automatically attained by electrolysis with the energy consumption of 0.435 kWh/m^3^. Figure 11B shows that the energy consumption was increased from 0.115 to 0.235 kWh/m^3^ with the cathodic potential decreasing from −1.0 to −1.4 V/SCE. We can infer that the pH-regulation took up most energy consumption (72%) in our system, and the whole system power consumption of per ton of wastewater was 0.605 kWh/m^3^, which translates into a cost of $0.04/m^3^ based on the average US industrial electricity rate ($0.0653/kWh). Iron sheet is very cheap and highly reusable ($1.4), which can counteract the cost. The cost of H_2_O_2_ was $0.16 with 0.6 L of consumption per ton of water, while the unit price of H_2_O_2_ was $0.27/L. Furthermore, *in situ* electrochemical synthesis of highly concentrated H_2_O_2_ (Yamanaka et al., 2003; Chen et al., 2017) could be used as a replacement for externally supplied H_2_O_2_ in the future, eliminating the chemical cost.

The specific energy consumption was calculated in terms of the removal of 1 kg of COD from PNP wastewater by the Fenton process (kWh/kg COD) using Eq. 13, where U, I, and t are the average voltage (V), applied current (A), and electrolysis time (s), respectively (Kurt et al., 2007; Zhao et al., 2016).

$$\text{SEC}=\frac{\text{U}\times \text{I}\times \text{T}}{\left({\text{COD}}_{0}\times {\text{V}}_{0}-{\text{COD}}_{\text{t}}\times {\text{V}}_{\text{t}}\right)\times 3.6}$$ (13)

After pH adjustment and the 30-min ICEF process, the PNP mineralization shows an SEC value of 4.92 kWh/kg COD, the results in this study demonstrated that the ICEF system was environmentally friendly, efficient, and inexpensive in comparison. It was proven that the operation of the system greatly enhanced the treatment of PNP wastewater. Compared to traditional Fenton and other electro-Fenton systems, the ICEF system has its own merits. Firstly, the pH-regulation divided electrolysis cell with the PTFE membrane as the separating material was simple and convenient compared to others, an acidic pH of 3.0–3.2 was automatically attained at a steady state within 1 min, which is suitable for most Fenton-like reactions (Babuponnusami and Muthukumar, 2014). In contrast, ∼60 min was required in the divided electrolysis cell using an ion exchange membrane as the separating material (Liu et al., 2007). Secondly, the cathode potential applied on the iron plate can decrease amounts of iron irons released under acid solution and precisely control the Fe (tot) production, avoiding the addition of iron salt and increase of water salinity. All these advantages together suggest that the ICEF system has potential for cost-effective and efficient degradation of recalcitrant organic pollutants. Future work should also focus on improving the efficiency of cathode reduction of ferric iron and improving the efficiency of H_2_O_2_ utilization.

## Conclusion

In this study, a novel eco-friendly iron cathode electrochemical Fenton (ICEF) system coupled with a pH-regulation divided electrolysis cell was developed for PNP degradation. In such a system, 100 mg/L of PNP was not only effectively degraded within 30 min (97%), but also efficiently mineralized with a COD and TOC removal efficiency of 79 and 60%, respectively. The optimal cathode potential exhibited strong inhibition on Fe (tot) production with the concentration of Fe (tot) significantly decreasing from 52 to 21 mg/L, minimizing the ferric sludge generation. And the conductivity increased slightly from 4.35 to 4.37 mS/cm, indicating that the present combined process negligibly affected the salt content of the wastewater. Notably, the system was inexpensive with an energy consumption of only 4.92 kWh/kg COD. In general, this study demonstrated that the present combined system is an effective and environmentally friendly technology for wastewater treatment.

## Data Availability

The original contributions presented in the study are included in the article/Supplementary Material, further inquiries can be directed to the corresponding author.
